# Evolution of TPS20-related terpene synthases influences chemical diversity in the glandular trichomes of the wild tomato relative *Solanum habrochaites*

**DOI:** 10.1111/j.1365-313X.2012.05040.x

**Published:** 2012-06-22

**Authors:** Eliana Gonzales-Vigil, David E Hufnagel, Jeongwoon Kim, Robert L Last, Cornelius S Barry

**Affiliations:** 1 Department of Horticulture, Michigan State UniversityEast Lansing, MI 48824, USA; 2 Department of Energy Plant Research Laboratory, Michigan State UniversityEast Lansing, MI 48824, USA; 3 Department of Plant Biology, Michigan State UniversityEast Lansing, MI 48824, USA; 4 Department of Biochemistry and Molecular Biology, Michigan State UniversityEast Lansing, MI 48824, USA

**Keywords:** specialized metabolism, glandular trichomes, chemical diversity, chemical ecology, terpene synthase, evolution, *Solanum habrochaites*

## Abstract

A systematic screen of volatile terpene production in the glandular trichomes of 79 accessions of *Solanum habrochaites* was conducted and revealed the presence of 21 mono- and sesquiterpenes that exhibit a range of qualitative and quantitative variation. Hierarchical clustering identified distinct terpene phenotypic modules with shared patterns of terpene accumulation across accessions. Several terpene modules could be assigned to previously identified terpene synthase (TPS) activities that included members of the TPS-e/f subfamily that utilize the unusual *cis-*prenyl diphosphate substrates neryl diphosphate and *2z,6z*-farnesyl diphosphate. DNA sequencing and *in vitro* enzyme activity analysis of TPS-e/f members from *S. habrochaites* identified three previously unassigned enzyme activities that utilize these *cisoid* substrates. These produce either the monoterpenes α-pinene and limonene, or the sesquiterpene 7-epizingiberene, with the *in vitro* analyses that recapitulated the trichome chemistry found *in planta*. Comparison of the distribution of *S. habrochaites* accessions with terpene content revealed a strong preference for the presence of particular *TPS20* alleles at distinct geographic locations. This study reveals that the unusually high intra-specific variation of volatile terpene synthesis in glandular trichomes of *S. habrochaites* is due at least in part to evolution at the *TPS20* locus.

## Introduction

Plants synthesize an array of specialized metabolites from diverse chemical classes known or hypothesized to provide selective advantage to the host within its ecological niche. These roles include direct and indirect defense against herbivores and insect pests, attraction of pollinators and seed dispersers, UV protection, and facilitation of beneficial symbioses (Li *et al.*, 1993; Landry *et al.*, 1995; Tewksbury and Nabhan, 2001; Wasson *et al.*, 2006; Degenhardt *et al.*, 2009; Unsicker *et al.*, 2009; Zhang *et al.*, 2009; Kang *et al.*, 2010; Klee, 2010; Klahre *et al.*, 2011; Rodriguez *et al.*, 2011; Weinhold and Baldwin, 2011). Many specialized metabolites are synthesized or accumulate in specific tissues or cell types. For example, glandular trichomes are epidermal appendages that occur on the surface of many plant species, which served as a first line of defense against insects and pathogens through the synthesis and storage of specialized metabolites, which included several used commercially as flavors, fragrances, and pharmaceuticals (Turner *et al.*, 1999; Sirikantaramas *et al.*, 2005; Covello *et al.*, 2007; Nagel *et al.*, 2008; Wang *et al.*, 2008; Xie *et al.*, 2008; Olsson *et al.*, 2009).

Glandular trichomes of the Solanaceae are structurally diverse and synthesize several classes of compounds including terpenoids, acyl sugars, phenylpropanoids, alkaloids and methylketones (Luckwill, 1943; van der Hoeven *et al.*, 2000; Kroumova and Wagner, 2003; Fridman *et al.*, 2005; Slocombe *et al.*, 2008; Schilmiller *et al.*, 2010a; Schmidt *et al.*, 2011). These compounds are implicated in plant defense against insect pests and pathogens, acting directly as toxins or repellents or indirectly through tritrophic interactions (Zhang *et al.*, 2008; Alba *et al.*, 2009; Bleeker *et al.*, 2009, 2011a; Weinhold and Baldwin, 2011). The economic importance of tomato, coupled with the availability of wild relatives that synthesize diverse glandular-trichome-derived metabolites has generated interest in utilizing exotic germplasm to improve insect resistance (Lawson *et al.*, 1997; Hartman and St Clair, 1999; Antonious *et al.*, 2005; Antonious and Snyder, 2006; Alba *et al.*, 2009; Lucatti *et al.*, 2010; Maluf *et al.*, 2010). *Solanum habrochaites* is a wild tomato species native to Southern Ecuador and Peru, which is characterized by an abundance of glandular trichomes that synthesize diverse insect repellent specialized metabolites, including terpenes (Weston *et al.*, 1989; Besser *et al.*, 2009; Bleeker *et al.*, 2009, 2011a; Sallaud *et al.*, 2009; Sifres *et al.*, 2011).

Terpenes are structurally diverse metabolites that are synthesized from the C5 compounds isopentenyl diphosphate (IPP) and dimethylallyl diphosphate (DMAPP) either from the plastid localized 2-C-methyl-d-erythritol 4-phosphate or the mevalonate pathways (Connolly and Hill, 1991; Chen *et al.*, 2011). There is tremendous plasticity in terpene biosynthesis in higher plants with a TPS enzyme frequently synthesizing multiple products from a single substrate (Tholl *et al.*, 2005; Martin *et al.*, 2010; Tholl and Lee, 2011). Furthermore, as plant genome sequences become available, it is increasingly evident that many *TPS* genes lie within clusters of tandemly duplicated genes that contain both functional and pseudogenes, a feature often indicative of rapid evolution that lead to phenotypic variation (Martin *et al.*, 2010; Chen *et al.*, 2011; Falara *et al.*, 2011).

The genome of the cultivated tomato *S. lycopersicum* contains approximately 44 *TPS* genes of which 29 are predicted to be functional and 18 have documented enzyme activity (Falara *et al.*, 2011). Several of these enzymes contribute to the trichome chemical phenotype (van der Hoeven *et al.*, 2000; van Schie *et al.*, 2007; Schilmiller *et al.*, 2009, 2010a). The majority of TPSs utilize the *trans* substrates GPP and *e,e-*FPP to synthesize monoterpenes and sesquiterpenes, respectively (Falara *et al.*, 2011). However, three members of the TPS-e/f subfamily, β-phellandrene synthase 1 from *S. lycopersicum* (SlPHS1) (TPS20), α-phellandrene synthase from *S. pennellii* (SpPHS1) and santalene/bergamotene synthase from *S. habrochaites* (ShSBS) were recently identified. These are chloroplast targeted and utilize the atypical *cisoid* substrates neryl diphosphate (NPP) and *2z,6z*-farnesyl diphosphate (*2z*,*6z*-FPP) to synthesize monoterpenes and sesquiterpenes, respectively (Sallaud *et al.*, 2009; Schilmiller *et al.*, 2009; Falara *et al.*, 2011). These three activities are encoded by highly conserved genes that reside at a single locus on chromosome 8, which suggested that they may represent orthologous loci (Sallaud *et al.*, 2009; Schilmiller *et al.*, 2009).

The unique features of terpene synthesis in glandular trichomes of tomato and its wild relatives prompted an analysis of the diversity of these isoprenoid compounds within *S. habrochaites*. The diversity of trichome-derived volatile mono- and sesquiterpenes in 79 accessions of *S. habrochaites* is described, which revealed qualitative and quantitative variation in the terpene complement. Hierarchical clustering placed the majority of these terpenes within distinct phenotypic modules whose biosynthesis could be traced to individual TPS enzymes. Much of the observed chemical diversity within *S. habrochaites* can be attributed to sequence variation within TPS20-related enzymes that utilize NPP and *2z*,*6z*-FPP as substrates. These enzymes synthesize several mono- and sesquiterpenes that included three newly identified enzymes that synthesize 7-epizingiberene, limonene and α-pinene. In addition, terpene profiles within *S. habrochaites* glandular trichomes are highly correlated with geographic distribution of the accessions.

## Results

### *Solanum habrochaites* accessions show high variability in their terpene composition

**Figure 1:**
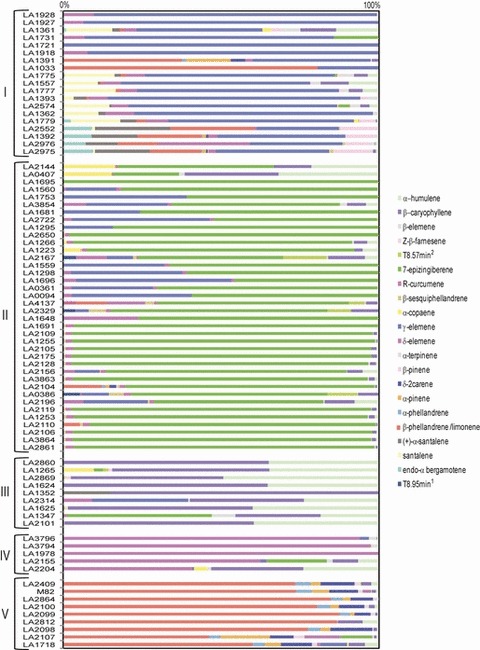
Terpene composition in glandular trichomes of leaves of *S. habrochaites*. The terpene profile of 79 *S. habrochaites* accessions and *S. lycopersicum* cv M82 is shown. Twenty-one analytical signals corresponding to distinct terpenes were identified. The terpene composition was expressed as % of total terpenes. Accessions are grouped according to Figure 2.

To explore the terpene diversity in the glandular trichomes of *S. habrochaites*, leaf dips of 79 accessions that cover the known geographic range of the species (Table S1) were screened by gas chromatography–mass spectrometry (GC–MS) (Schilmiller *et al.*, 2010a). A total of 21 analytical signals were identified that corresponded to six monoterpenes, 13 sesquiterpenes and two unknown compounds (Figure 1 and Table S2). These data reveal that some accessions possess a relatively simple terpene composition dominated by a single major compound e.g. LA2109 (group II, with 7-epizingiberene as a major component) and LA1978 (group IV, with δ-elemene) whereas others exhibit more complex profiles, e.g. LA2975 (group I) and LA2107 (group V).

**Figure 2:**
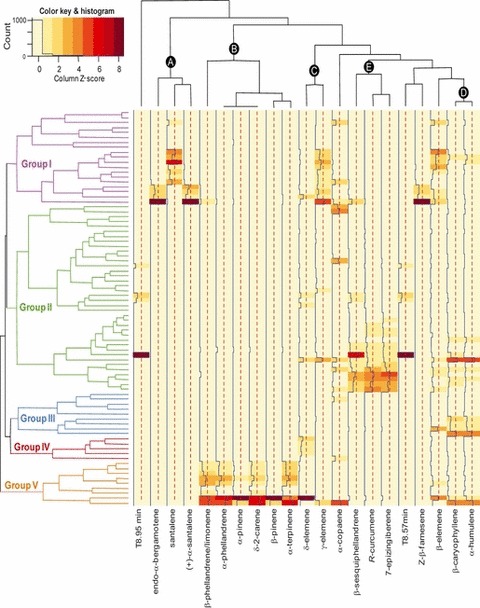
Two-way cluster analysis of normalized peak areas from *S. habrochaites* accessions. The peak areas normalized to the internal standard and leaf dry weight were calculated for each of the 21 terpene signals in 79 *S. habrochaites* accessions and M82. The average from two to three replicates was calculated and used for construction of the heatmap. Each row represents the average terpene composition for an accession, and the dendrogram on the left represents the clustering of accessions based on chemical profile. Accessions from the same group (I through V) have similar terpenes. Each column represents the abundance of a particular terpene in all accessions, and the dendrogram representing the clustering of terpenes based on presence/absence in the accessions is shown at the top. Terpenes from the same module (A through E) show similar patterns of accumulation across accessions. Clustering was performed as described in Experimental procedures. The distance of the blue trace line from the center of each cell (red dotted line) is proportional to the abundance of the compound. The peak areas were centered in the column direction. T 8.57 min and T 8.95 min represent unidentified terpenes eluting at 8.57 and 8.95 min, respectively. The order of the accessions in the dendrogram is the same as those presented in Figure 1.

Peak area data normalized to the internal standard and leaf dry weight were used for hierarchical clustering (Figure 2). The presence or absence of analytical signals was used to cluster terpenes to identify distinct phenotypic modules composed of terpenes whose accumulation is correlated across accessions. Four of these modules were assigned to previously identified TPS activities in *Solanum* trichomes. For example, module A includes endo-α-bergamotene and (+)-α-santalene, products of ShSBS previously characterized from *S. habrochaites*LA1777 (Sallaud *et al.*, 2009). Module B consists of six monoterpenes, five of which are the products of SlPHS1 from *S. lycopersicum* (Schilmiller *et al.*, 2009). Module C includes δ-and ε-elemene, the Cope rearrangement products of germacrene C and B, which are synthesized by TPS9 (Colby *et al.*, 1998). Finally, module D includes the products of TPS12, β-caryophyllene and α-humulene (Schilmiller *et al.*, 2010a; Bleeker *et al.*, 2011b; Falara *et al.*, 2011). Collectively, these observations support the hypothesis that terpenes within a module are produced by the same TPS(s).

A dendrogram was constructed to cluster accessions according to similarity in terpene content. Five chemical groups (group I through V) that were stable through several iterations of the algorithm were defined, which revealed patterns of terpene distribution across the accessions (Figure 2). Group I, which contains the previously characterized LA1777 (Figures 1 and 2), is defined by high abundance of γ-elemene. Group I could be further divided in two subgroups. Accessions from the first subgroup contain sesquiterpenes synthesized by ShSBS (module A) (e.g. LA1777 and LA1393). The second subgroup also has γ-elemene as the major terpene, but lacks terpenes made by ShSBS (e.g. LA1721). Group II, which constitutes the largest group of 38 accessions is defined by the presence of 7-epizingiberene as the major peak (Bleeker *et al.*, 2011a). The presence of 7-epizingiberene is highly correlated with the presence of *R*-curcumene and β-sesquiphellandrene, and this chemical phenotype is collectively designated module E. Group V accessions, which also include *S. lycopersicum* M82, produce a mixture of monoterpenes in which β-phellandrene is the major compound together with smaller amount of limonene, α-pinene, δ-2-carene, α-terpinene and α-phellandrene. β-Caryophyllene and α-humulene are the major compounds identified in nine accessions from group III, whereas δ-elemene is the prominent compound in five accessions from group IV. These compounds are not specific to a single group but are distributed across multiple groups. In contrast, module A is exclusive to group I, module B is mostly present in group V, and module E predominates in group II. Together, results of these analyses simplify the considerable terpene diversity within *S. habrochaites* trichomes, which suggested common biosynthetic origins that may be reflected in the activity of relatively few TPS activities.

### Variation in terpene quantity is associated with terpene composition

A subset of 27 accessions that represent the diversity from the larger dataset were selected and re-grown both to validate the original data derived from 79 accessions and quantify the volatile terpene levels using β-caryophyllene and γ-terpinene standards (see Experimental procedures). The total amount of mono- and sesquiterpenes in this subset of accessions ranged 100-fold from 171 μg g^−1^ dry weight in LA1265 to 19 200 μg g^−1^ dry weight in LA2106 (Figure S1). There were differences in terpene quantity between groups (anova*F*: 4.753, *P*-value < 0.01). In addition, a Tukey Honest Significant Differences test revealed that group II means were significantly different from those of group I and III at a *P*-value < 0.05, due to the high accumulation of 7-epizingiberene in group II accessions. In contrast, the abundance of β-caryophyllene and α-humulene did not vary significantly across the five groups.

### Phylogenetic analysis of TPS20-related homologs from *S. habrochaites*

Three TPS-e/f subfamily enzymes, *SlPHS1* (*SlTPS20*), *SpPHS1* and *ShSBS*, contribute to chemical variation in *S. habrochaites*LA1777, *S. lycopersicum* M82 and *S. pennellii*LA0716 trichomes (Sallaud *et al.*, 2009; Schilmiller *et al.*, 2009; Falara *et al.*, 2011). Based on the diverse catalytic activities and the strong level of sequence conservation between these enzymes, we hypothesized that sequence variation within TPS20-related enzymes could contribute to the observed terpene diversity in *S. habrochaites*. Primers designed from *SlPHS1* were used to amplify full-length cDNAs from 23 *S. habrochaites* accessions that led to the recovery of 34 *TPS* cDNAs that represented 22 non-redundant sequences. In several accessions distinct cDNAs were recovered that may reflect the presence of separate, closely related loci, but could also be due to heterozygosity which is widespread within *S. habrochaites* (Rick *et al.*, 1979). The cDNAs encoded predicted proteins of either 777 or 778 amino acids that contain predicted chloroplast targeting sequences together with modified versions of the ‘DDXXD’ (in most of the sequences, the third aspartic acid is replaced by a glutamic acid) and NSE/DTE motifs found in TPSs (Figure S2) (Martin *et al.*, 2010). Phylogenetic studies suggest that all known TPSs are derived from diterpene synthases involved in gibberellin biosynthesis (Chen *et al.*, 2011). While most plant TPSs are shorter than diterpene synthases, the sequences recovered here and others from TPS-e/f have maintained the internal sequence element or ‘γ domain’ (Hillwig *et al.*, 2011).

**Figure 3:**
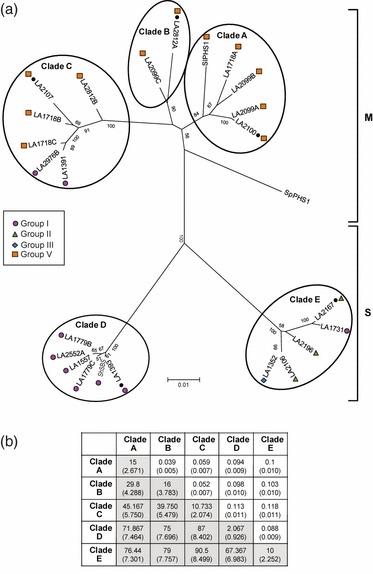
Phylogenetic relationship of TPS20-related sequences from *S. habrochaites*. (a) An unrooted tree constructed with the ME method using 25 predicted protein sequences, representing a total of 37 sequences from various *S. habrochaites* accessions (identical sequences at the nucleotide level were eliminated to avoid overcrowding the tree) together with SlPHS1 (FJ797957), SpPHS1 (JN412071), and ShSBS (ACJ38409). Two major groups designated M and S, subdivide into clade A, B and C; and clade D and E, respectively. Bootstrap values above 50 are shown. Colored symbols indicate the chemical group (defined in Figure 2) from which each sequence was isolated. Sequences chosen for codon optimization and expression in *E. coli* are highlighted (•). (b) Estimates of average evolutionary divergence. Shaded, the number of amino acid differences per sequence from averaging over all sequence pairs within and between each group is shown. White, the p-distance (1-amino acid identity) from averaging over all sequence pairs between groups is shown. Standard error estimates are shown in parentheses. The analysis involved 24 amino acid sequences, corresponding to the five clades shown in (a). Positions containing gaps and missing data were eliminated. Analyses were conducted in MEGA5.

Phylogenetic analysis of the *S. habrochaites* amino acid variants and previously characterized enzymes SlPHS1, *S. pennellii* PHS1 (SpPHS1), and ShSBS revealed separation into two major clusters: M and S (for Monoterpenes and Sesquiterpenes, respectively, see below) (Figure 3 and Figure S3). As expected from the similarity of the chemical profiles, the sequences obtained from group V accessions cluster with SlPHS1 (cluster M in Figure 3). This finding indicates that the *S. habrochaites* sequences obtained from monoterpene-producing accessions have higher similarity to *SlPHS1* than to other *S. habrochaites* sequences from cluster S. Cluster M is further divided into clade A (more closely related to SlPHS1) and clades B and C. Clade C sequences have an in-frame 3-bp deletion at alanine-538, which is adjacent to the DDXXE motif (Figure S2). This clade includes sequences obtained from groups I and V. However, the accessions that belong to group I, namely LA2976 and LA1391, synthesize monoterpenes such as β-phellandrene and limonene as do other accessions from group V (Figure 1).

Cluster S is composed of sequences that are more similar to ShSBS from LA1777, and are subdivided into clades D and E. Clade D consists of accessions that belong exclusively to group I. Notably, this clade displays the highest level of sequence conservation among members, with a mean amino acid difference between sequence pairs of two, compared with 15, 16, 11 and 10 in clades A, B, C and E, respectively (Figure 3b). Clade E includes accessions from groups I, III, and IV, but predominantly from II.

### A genomic rearrangement contributes to *TPS-e/f* sequence divergence

**Figure 4:**
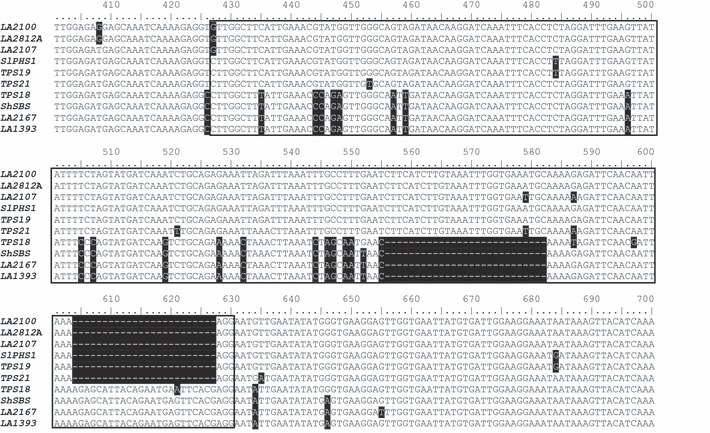
Evidence for a genomic rearrangement within exon 4. (a) Comparison of the nucleotide sequence of the *TPS-e/f* family members LA2100, LA2812A, LA2107, LA2167, LA1393, and *ShSBS*, together with *TPS18* (JN412088)*, 19* (JN412072)*, SlPHS1* (FJ797957) and *21* (JN412087) from *S. lycopersicum*. Non-identical residues are highlighted in black. Exon 4 is denoted by a box.

**Figure 5:**
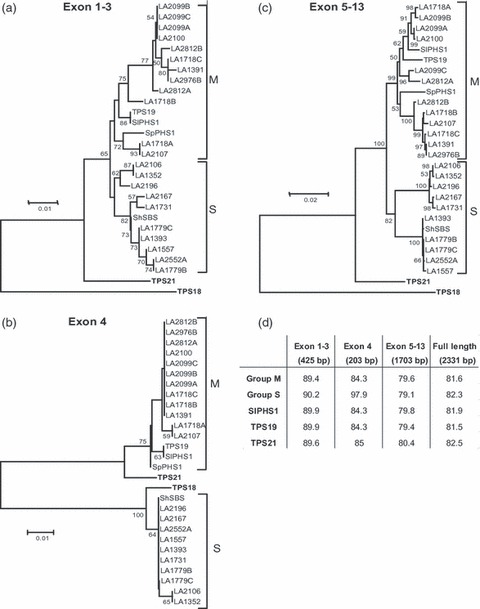
Sequence exchange between *TPS20* and *TPS18* contributes to *TPS* diversity. (a–c) Phylogenetic analysis of *TPS-e/f* nucleotide sequences. A codon alignment performed with MUSCLE was used to reconstruct the phylogeny of the *TPS-e/f* subfamily with sequences from *S. habrochaites* together with *TPS18* (JN412088), *TPS19* (JN412072), *TPS21* (JN412087), *SlPHS1* (FJ797957), *SpPHS1* (JN412071), and *ShSBS* (FJ194970). The alignment was divided into three segments corresponding to exons 1 through 3, exon 4, and exons 5 through 13 before tree reconstruction with an ME model in MEGA5. (d) Percent nucleotide identity of *TPS-e/f* sequences compared to *TPS18* from *S. lycopersicum*. A codon alignment was divided into three regions as above. Nucleotide identity between *TPS18* from *S. lycopersicum* and each *S. habrochaites* sequence was calculated, and the average from each group obtained. For comparison, the nucleotide identity of *SlPHS1*, *TPS19* and *TPS21* is shown.

The sequence divergence within the *S. habrochaites* TPS-e/f family raises questions about the evolutionary origins of this diversity. To investigate this situation further, a codon alignment was performed that included the sequences of three additional TPS-e/f subfamily members identified within the *S. lycopersicum* chromosome 8 cluster; *TPS18, 19* and *21* (Figure 4) (Falara *et al.*, 2011). These were included because TPS20 (*SlPHS1*) is 82, 98.5 and 93% identical at the nucleotide level to *TPS18, 19* and *21* respectively, which suggested that they arose from a common ancestor by tandem duplication and functional divergence (Falara *et al.*, 2011). Inspection of the alignment reveals that all sequences from group S contain a region of 200 bp (boxed region, Figure 4), which corresponded to exon 4 in *SlPHS1*, which is more similar to *TPS18* than to other sequences from group M. To illustrate this result, phylogenetic analysis of the codon alignments was performed following separation of the coding sequences into three regions, exons 1–3, exon 4, and exons 5–13 (Figure 5a–c). The topology of the tree constructed with exon 4 varied considerably compared with the trees derived from the other regions. Within the exon 4 region, the sequences from group S cluster together with *TPS18* from *S. lycopersicum*, whereas in the other regions, *TPS18* forms the out group for all sequences. In exon 4 the average identity of group S to *TPS18* is 98% at the nucleotide level, compared with 90 and 79% in the other two regions (Figure 5d). In contrast, *S. habrochaites* sequences from group M are 84% identical to *TPS18* in exon 4, and 89 and 80% identical in the other two regions (Figure 5d). These observations suggest that a form of genomic rearrangement has occurred within exon 4 of *S. habrochaites* accessions from group S, which introduced a sequence more similar to *TPS18* that contributes to sequence divergence within the *TPS-e/f* subfamily.

### Plasticity in the activity of TPS20-related enzymes from *S. habrochaites*

SlPHS1 and ShSBS share 91% amino acid sequence identity, yet they use the structurally distinct NPP and *2z,6z-*FPP for the synthesis of mono- and sesquiterpenes, respectively (Sallaud *et al.*, 2009; Schilmiller *et al.*, 2009). To explore the hypothesis that sequence diversification has resulted in functional divergence at TPS20 within *S. habrochaites*, codon optimized versions (Ikemura, 1981) of representatives from each of the five major clades in the phylogenetic tree were expressed in *E. coli*.

**Figure 6:**
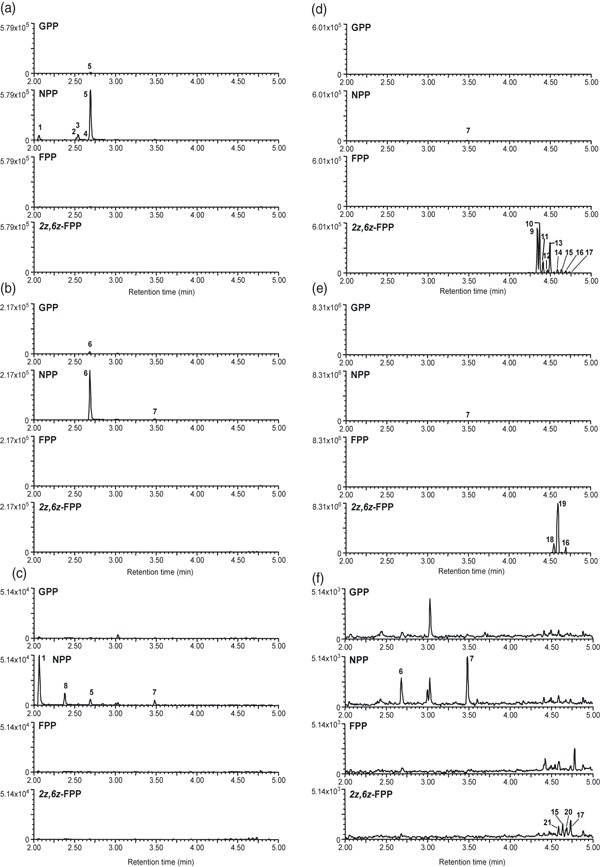
Headspace collection of volatiles produced by recombinant TPS from LA2100 (ShPHS1) (a), LA2812A (ShLMS) (b), LA2107 (ShPIS) (c), LA1393 (ShSBS) (d) and LA2167 (ShZIS) (e) expressed in *E. coli* and assayed with GPP, NPP, FPP or *2z,6z*-FPP as substrates. Reactions with the empty vector are included as a control (f). Extracted ion chromatograms for *m/z* 93 are shown. 1, α-pinene; 2, δ-2-carene; 3, α-phellandrene; 4, α-terpinene; 5, limonene and β-phellandrene; 6, limonene; 7, α-terpineol; 8, β-pinene; 9, endo-α-bergamotene; 10, (+)-α-santalene; 11, (−)-exo-α-bergamotene; 12, (−)epi-β-santalene; 13, (+)-endo-β-bergamotene; 14, (*Z*)-β-farnesene; 15, β-bisabolene; 16, β-sesquiphellandrene.; 17, unknown; 18, *R*-curcumene; 19, 7-epizingiberene; 20, unknown; 21, unknown. A series of chromatograms with improved resolution of low abundance peaks obtained using *2z,6z*-FPP as a substrate is provided in Figure S6. Note that peaks 15, 17, 20 and 21 appear to be non-specific products.

The recombinant proteins were tested with each of the potential substrates: geranyl diphosphate (C10, GPP), *e,e-*farnesyl diphosphate (C15, *e,e-*FPP), and the *cisoid* substrates neryl diphosphate (C10, NPP) and *2z,6z-*farnesyl diphosphate (C15, *2z,6z-*FPP). The predicted proteins from group M utilized NPP to synthesize a variety of monoterpenes. As predicted based on sequence similarity, the recombinant enzyme from clade A accession LA2100 produced a monoterpene profile indistinguishable from SlPHS1 (Schilmiller *et al.*, 2009), with β-phellandrene as the major product, along with limonene, α-pinene, δ-2-carene, α-phellandrene and γ-terpinene (Figure 6a). The products of this enzyme (designated ShPHS1) with NPP substrate match the terpene profile from trichome extracts of accession LA2100 (Figure S4a). In contrast, the enzyme LA2812A from clade B (one of the sequences recovered from accession LA2812) converted NPP to limonene, which again matches the profile observed in extracts of LA2812 trichomes and is therefore designated ShLMS (Figure 6b, Figures S4b and S5). The enzyme from clade C accession LA2107 converted NPP predominantly to α-pinene together with lower amounts of β-pinene and β-phellandrene and/or limonene (Figure 6c), and therefore is named ShPIS. Trichomes from LA2107 also accumulate the compounds produced by ShPHS1; however the presence of high β-pinene levels in the trichomes can only be explained by the activity of ShPIS (Figure S4c). In general, enzymes from the sesquiterpene-rich clades D and E (from accessions LA1393 and LA2167, respectively) did not produce detectable amounts of monoterpenes from GPP or NPP except for very low amounts of limonene and α-terpineol in assays that contained NPP (Figure 6d, e). However, these compounds were also observed in reactions that contained the empty vector and NPP and, in the case of limonene, are at least two orders of magnitude lower than in reactions that contained ShLMS (Figure 6b,f). Furthermore, α-terpineol was not detected in trichome samples (Figure 1 and Figure S4). Therefore, it is likely that the formation of limonene and α-terpineol can occur either enzymatically or non-enzymatically in the crude *E. coli* extracts used to perform the enzyme assays.

The enzyme from clade D produced a sesquiterpene profile identical to that made by the closely related LA1777 ShSBS when supplied with *2z,6z-*FPP as substrate (Figure S4d). In contrast, the enzyme from clade E utilized *2z,6z-*FPP to synthesize 7-epizingiberene, *R*-curcumene and β-sesquiphellandrene, the module E terpenes (Figure 2 and Figure S4e). The enzyme therefore is designated ShZIS.

In conclusion, the functional characterization of five different alleles of TPS20 from *S. habrochaites* revealed five distinct enzyme activities. Enzymes that belong to group M utilize NPP for the production of monoterpenes, whereas enzymes from group S utilize *2z,6z-*FPP to synthesize sesquiterpenes. Collectively, these enzymes synthesize many of the major terpenes identified in the trichomes of *S. habrochaites*.

### A geographical-climatic context for terpene diversity

**Figure 7:**
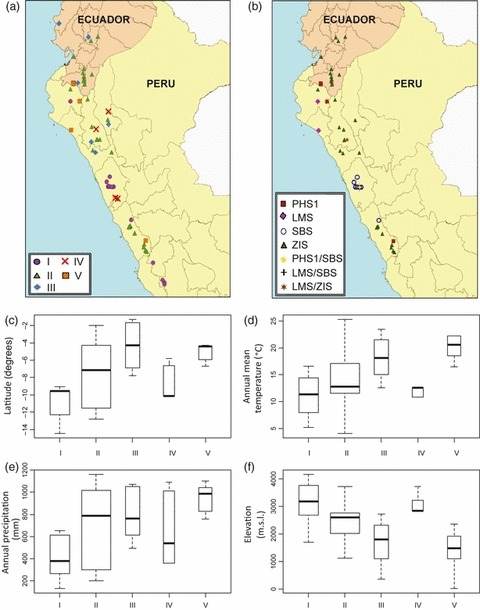
Geographical distribution of *S. habrochaites* accessions and climatic data. (a, b) Map of Ecuador and Peru showing the collection coordinates of *S. habrochaites* accessions. (a) Accessions were classified according to the chemical groups determined by hierarchical clustering (Figure 2). (b) Accessions were classified based TPS-e/f derived terpenes. (c–f) Box plots displaying differences between chemical groups in climate data of collection sites. The box plots show lower and upper quartiles, the extreme values that are not outliers and the median. Outliers have been omitted. Boxes are drawn with widths proportional to the square-roots of the number of observations in the group. Maps and climate data were obtained in DIVA-GIS. Box plots were constructed using R.

The *S. habrochaites* accessions used in this study were collected in Peru and Ecuador from 1948 to 1997, and the geographical coordinates of most of the collection sites are available (http://tgrc.ucdavis.edu/). This information was used to examine the terpene variation of *S. habrochaites* throughout its range of distribution. The accessions were classified according to the five chemical groups described in Figure 2 and mapped using their geographical coordinates (Figure 7a,c). Additionally, annual mean temperature, annual precipitation and elevation were calculated for each group (Figure 7d–f).

Co-occurrence of the terpene groups with the geographical distribution of the accessions is observed. Group I accessions were preferentially located in the higher altitude southern range of the distribution, with lower mean temperatures and annual precipitation, whereas groups III and V were located further north, in regions that are at lower elevation, and warmer with a more humid climate. In contrast, group II accessions are present over a wide area of the distribution.

A similar analysis was conducted to classify the accessions based on whether their chemical profile contains the predominant terpenes made by members of the TPS20-related enzymes, namely β-phellandrene, α-pinene and δ-carene for ShPHS1, limonene for ShLMS, santalene/bergamotene for ShSBS, or 7-epizingiberene for ShZIS (Figure 7b and Table S1). The production of santalene and bergamotene is confined to Ancash and Lima in Peru (Figure 7b and Table S1), whereas accessions from group I that lack the products of ShSBS are preferentially located in the southernmost area of the distribution. The narrow distribution of these terpenes is consistent with the low evolutionary divergence found in the sequences recovered from clade D that encode ShSBS enzymes (Figure 3b). A different situation is observed for the β-phellandrene/limonene-producing accessions, which were mainly collected in the border between Ecuador and Peru, yet clade A possessed large sequence divergence (Figure 3b). Meanwhile, the production of 7-epizingiberene is widely distributed and alleles from clade E showed intermediate levels of sequence divergence. Notably, the distribution of accessions that synthesize 7-epizingiberene is not continuous as they are not located in the area where santalene-/bergamotene-producing accessions predominate. Furthermore, no sequence features were detected within clade E that distinguishes the accessions from the southern region of the distribution from those in the northern region that are separated by the santalene- and bergamotene-producing accessions (data not shown).

## Discussion

### *Solanum habrochaites* accessions differ in the type and quantity of trichome derived terpenes

**Figure 8:**
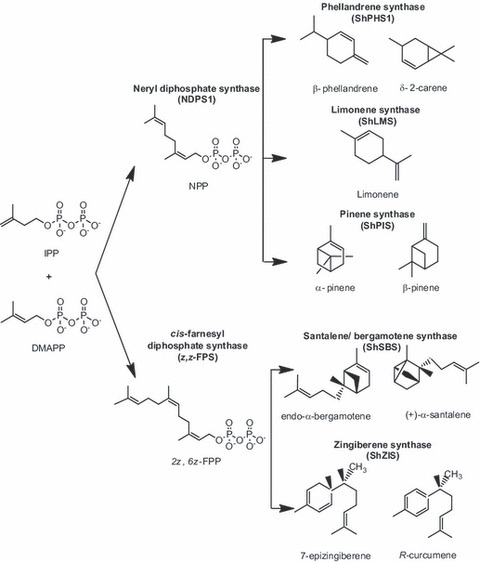
Terpene biosynthesis in *S. habrochaites* trichomes using the *cisoid* substrates NPP and *2z,6z*-FPP.

The volatile terpene profiles in the trichomes of 79 accessions of *S. habrochaites* are qualitatively and quantitatively diverse (Figure 1 and Figure S1). Most accessions produce predominantly sesquiterpenes, which included 7-epizingiberene, *R-*curcumene, endo-α-bergamotene and (+)-α-santalene, which are not present in cultivated *S. lycopersicum* M82 or within 53 additional *S. lycopersicum* accessions that we screened (unpublished data). However, a small group of accessions, mainly in group V, resemble cultivated tomato in that they synthesize predominantly monoterpenes with a profile dominated by β-phellandrene (Figure 1 and Figure S1b) (Schilmiller *et al.*, 2009). Moreover, three *S. habrochaites* TPSs (ShPHS1, ShPIS and ShLMS) convert NPP to monoterpenes; each produces an *in vitro* terpene profile that matches trichome extracts (Figures 6, 8 and Figure S4). Together, these data suggest the existence of a prenyltransferase in select accessions of *S. habrochaites* that is similar to the NPP-producing *NDPS1* from *S. lycopersicum* (Schilmiller *et al.*, 2009).

Hierarchical clustering analysis reveals close association between specific terpenes in different *S. habrochaites* accessions, leading to the hypothesis that these modules are produced by specific TPSs. In fact, four terpene modules (A–D) correspond to products made by previously characterized *Solanum* TPSs (Colby *et al.*, 1998; Sallaud *et al.*, 2009; Schilmiller *et al.*, 2009, 2010a; Bleeker *et al.*, 2011b; Falara *et al.*, 2011), and a fifth, characterized by the presence of 7-epizingiberene, are products of ShZIS (Figures 2 and 6). Quantitative variation in terpene levels also correlates with qualitative composition (Figure S1). In general, 7-epizingiberene-producing accessions in group II accumulate higher levels of volatile terpenes than the other groups. In contrast, the products of module A, santalene, (+)-α-santalene and endo-α-bergamotene, which are synthesized by ShSBS, represent minor compounds in group I accessions (Figure S1). This difference in abundance presumably is caused by further metabolism of the ShSBS products to their corresponding acids (Frelichowski and Juvik, 2001, 2005; Besser *et al.*, 2009).

### The central role of TPS20-related enzymes in trichome chemical diversity

Previous studies highlighted the importance of TPS20-related enzymes in terpene biosynthesis in the glandular trichomes of tomato and related wild species (Sallaud *et al.*, 2009; Schilmiller *et al.*, 2009; Falara *et al.*, 2011). Furthermore, analysis of nearly isogenic lines that contain genomic regions from LA1777 in a *S. lycopersicum* background demonstrated that santalene/bergamotene and monoterpene accumulation are controlled by a single locus or tightly linked loci and subsequent identification of *SlPHS1* and *ShSBS* showed that these genes lie within orthologous regions on chromosome 8 (van der Hoeven *et al.*, 2000; Besser *et al.*, 2009; Sallaud *et al.*, 2009; Schilmiller *et al.*, 2009; Falara *et al.*, 2011). These data led us to hypothesize that additional TPS20-related enzymes contribute to the terpene diversity encountered in *S. habrochaites* trichomes. Homology-based cloning, coupled with phylogenetic analysis and enzyme assays with recombinant proteins, support the hypothesis. Heterologous expression in *E. coli* revealed that sequences obtained from LA2100 (ShPHS1), LA2812 (ShLMS) and LA2107 (ShPIS) encode monoterpene synthases that convert the C10 substrate NPP to a blend of terpenes that resembles the profiles observed *in planta* (Figure 8 and Figure S4). Similarly, the TPS20-related enzymes from accessions LA1393 (ShSBS) and LA2167 (ShZIS) synthesize mixtures of sesquiterpenes from *2z,6z*-FPP (Figure 8).

The location of genes involved in plant specialized metabolism, which included terpene biosynthesis, within gene clusters is an emerging paradigm and creates the potential for tandem duplications, deletions and rearrangements, possibly generating phenotypic variation (Martin *et al.*, 2010; Chu *et al.*, 2011; Falara *et al.*, 2011). *SlPHS1* (*TPS20*) is part of a gene cluster that contained three additional *TPS-e/f* subfamily members of unknown function, *SlTPS18*, *SlTPS19* and *SlTPS21* (Falara *et al.*, 2011). *SlPHS1* (*TPS20*) is especially closely related to *SlTPS19* and these genes likely arose through recent gene duplication (Falara *et al.*, 2011). The *TPS20-*related sequences identified in this study are more closely related to *SlPHS1* and *SlTPS19* than to *SlTPS18* and *SlTPS21* (Figures 4 and 5). However, *SlPHS1* and *SlTPS19* are more similar to each other than they are to any of the *S. habrochaites TPS20*-related sequences. Therefore, these relationships render assessment of orthology and paralogy inconclusive at the current level of resolution. Resolution of orthology and paralogy will require analysis of the relative placement of these genes in chemically diverse *S. habrochaites* accessions to resolve potential tandem gene duplications or additional rearrangements within the gene cluster. Indeed, evidence of a gene rearrangement event was detected that led to incorporation of a sequence derived from *SlTPS18* into the clade S genes that encode ShSBS and ShZIS (Figures 4 and 5). The nature of this rearrangement is unknown but may have arisen through a gene conversion event or an alteration in splicing and lead to a change in the exon/intron boundary position (Innan and Kondrashov, 2010). Distinguishing these possibilities will require sequencing of the corresponding genomic loci.

Some of the observed chemical variation in *S. habrochaites* may be caused by heterozygosity within accessions that result from self-incompatibility and subsequent cross-pollination (Rick *et al.*, 1979). In addition, many of the accessions were collected from more than a single individual, which increased the likelihood that multiple alleles are present within a given seed lot (http://tgrc.ucdavis.edu/). For example, we commonly found plants that produce terpenes characteristic of two TPS20-related enzymes (Table S1 and Figure S7), which suggested either the expression of paralogous genes or heterozygosity within the terpene gene cluster on the top of chromosome 8. Consistent with these diverse chemical phenotypes, more than one *TPS20*-related sequence was recovered from the same accession on several occasions (Figure 3, accession numbers followed by letters). In addition, many of the accessions were collected from more than a single individual, which increased the likelihood that multiple alleles are present within a given seed lot (http://tgrc.ucdavis.edu/). This situation was observed through the recovery of three TPS20-related alleles from LA1718, LA1779 and LA2099 (designated A through C) (Figure 3 and Figure S3). While the alleles recovered from LA1718 and LA2099 may encode separate monoterpene synthases, the high sequence similarity of the three alleles from LA1779 suggests that all encode a functional SBS (Figure 3 and Figure S3). Furthermore, it is documented (http://tgrc.ucdavis.edu/) that the sample size for this accession was two individuals. Therefore, if three alleles were present in these two individuals, it is possible that they would have been maintained in subsequent seed lots. However, while it is clear that considerable heterogeneity is present within *S. habrochaites*, it is noteworthy that no accessions were identified that synthesize the products of both ShSBS and ShZIS. The significance of this result remains unclear but may be due to restricted geographic distribution of the ShSBS that contained accessions leading to reproductive isolation (Figure 7b).

### Some accessions of *S. habrochaites* lack terpenes derived from *cisoid* isoprenoid diphosphate substrates

The glandular trichomes of *Solanum* spp. possess the ability to utilize both the cytosolic mevalonate and plastidic 2-C-methyl-d-erythritol 4-phosphate pathways to synthesize precursors for terpene biosynthesis (Colby *et al.*, 1998; van der Hoeven *et al.*, 2000; Sallaud *et al.*, 2009; Schilmiller *et al.*, 2009, 2010b). In addition, the use of the plastid localized 2-C-methyl-d-erythritol 4-phosphate pathway to synthesize the *cisoid* substrates NPP and *2z,6z*-FPP has thus far only been reported in *Solanum* trichomes (Figure 6 and Figure S4) (Sallaud *et al.*, 2009; Schilmiller *et al.*, 2009; Falara *et al.*, 2011). However, most *S. habrochaites* accessions from groups III and IV are exceptions in that they do not synthesize terpenes from *cisoid* substrates. In contrast, they accumulate β-caryophyllene/α-humulene and γ-elemene/δ-elemene, the products of SlTPS12 and SlTPS9, respectively that are synthesized in the cytosol from *trans e,e-FPP* (Figure 1) (Colby *et al.*, 1998; van der Hoeven *et al.*, 2000; Schilmiller *et al.*, 2010b; Falara *et al.*, 2011). Similarly, group I can be divided into a subgroup that contains the sesquiterpene products of ShSBS and a subgroup that does not (Figure 2). Furthermore, accessions from groups III and IV generally accumulate lower levels of volatile terpenes than groups that utilize the *cisoid* substrates for terpene biosynthesis (Figure S1). Sequestering a proportion of volatile terpene biosynthesis in the chloroplast and utilizing a separate substrate pool creates the opportunity to increase terpene synthesis and generate variation in the terpene profile of glandular trichomes.

Several possibilities exist that could explain the lack of *cis* substrate-derived terpenes in accessions from groups I, III, and IV. For example, *TPS20*-related genes may not be expressed in trichomes, are absent, or enzymatically non-functional. Evidence that supported this result derives from failed attempts to clone *TPS20*-related cDNAs from several of these accessions (e.g. LA2101). Second, the substrate for TPS may be unavailable due to lack of expression or mutation in a *cis*-prenyltransferase. This finding is supported by the observation that several accessions that do not accumulate 7-epizingiberene (LA2860, LA1625 and LA3794) contain an apparently functional enzyme that is identical to that recovered from 7-epizingiberene-producing accessions (LA2104 and LA2106). Additional characterization of the chromosome 8 TPS cluster from select group I, III and IV accessions will provide insight into these phenomena.

### The evolution of monoterpene and sesquiterpene biosynthesis is driven by variation in *TPS20*-related genes in *S. habrochaites*

The data presented suggest that the diversity observed in terpene production derived from TPS20-related enzymes in *S. habrochaites* trichomes can be explained by a small number of molecular events. In this model, before the split of *S. lycopersicum* and *S. habrochaites*, a member of the TPS-e/f subfamily, likely a diterpene synthase, acquired the ability to use NPP to synthesize monoterpenes. This finding is consistent with the finding that *S. pennellii* trichomes also utilize NPP to produce monoterpenes (Falara *et al.*, 2011). Additionally, the large sequence divergence within cluster M supports the hypothesis that a monoterpene synthase is the ancestral form of the enzyme (Figure 3). Moreover, monoterpene-producing accessions are preferentially located in the border region between Peru and Ecuador, a hotspot for Solanaceae diversity and the proposed origin of *S. habrochaites* (Sifres *et al.*, 2011). Subsequently, the appearance of a TPS that could use *2z,6z-*FPP instead of NPP could have arisen by introduction of the *TPS18* exon four region through gene conversion or other genomic rearrangement (Figures 4 and 5). Although the impact of the exon 4 region from *TPS18* on enzymatic activity has not been directly demonstrated, its association with sesquiterpene-producing enzymes is consistent with the hypothesis that it has a role in changing the substrate specificity, allowing the enzyme to accept *2z,6z-*FPP as a substrate. This rearrangement occurred prior to the split of *ShZIS* and *ShSBS*. Furthermore, it is likely that *ShZIS* originated before *ShSBS* as the former has higher evolutionary divergence and a wider geographic distribution (Figures 3 and 7b). It is also possible that the ability to synthesize santalene and bergamotene provides a strong fitness advantage, for example tolerance of insect herbivores or pathogens, which created strong positive selection for *ShSBS* in central Peru.

Field experiments are required to test the adaptive value of each *TPS20-*related gene under its native environment. This approach would help establish the biotic factors that influence chemical variation within *S. habrochaites*. Similarly, the identification of ShZIS provides an opportunity to breed or engineer the biosynthesis of 7-epizingiberene, a known insect repellent (Bleeker *et al.*, 2011a), in trichomes or other tissues of *S. lycopersicum* and additional crop plants for improved arthropod resistance.

## Experimental procedures

### Plant material and growth conditions

Seeds from 79 accessions of *Solanum habrochaites* and *Solanum lycopersicum* cv. M82 (LA3475) were obtained from the C.M. Rick Tomato Genetics Resource Center (TGRC) (http://tgrc.ucdavis.edu/) (Table S1). Seeds were germinated on filter paper in the dark. After germination, two to three plants per accession were grown on Jiffy-7 Peat Pellets (http://www.hummert.com/) for 3 weeks as previously described (Schilmiller *et al.*, 2009). Plants were transplanted into peat-based compost supplemented with fertilizer in greenhouses equipped with environmental controls and supplemental lighting at Michigan State University, East Lansing, MI, USA.

### Metabolite extraction and terpene analysis

Three-week-old plants were used for terpene analysis. Briefly, a leaflet from the second newly emerging leaf was dipped in 1 ml of methyl *tert*-butyl ether that contained 5 ng μl^−1^ of tetradecane as an internal standard, and allowed to rock for 1 min. GC–MS analysis was performed as previously described (Schilmiller *et al.*, 2009). The data were exported into MassLynx V4.1 and quantification performed with QuanLynx (http://www.waters.com/). The extracted ion chromatogram was obtained at *m/z* 93, which is common to all mono- and sesquiterpenes. Peaks with an area >5 in at least one accession were quantified. This criterion narrowed the list of peaks identified; however, minor peaks did not have a reliable mass spectrum for peak identification, nor comparison across multiple samples. The resulting relative abundance for each terpene in a sample was normalized to the internal standard and leaflet dry weight. Terpene identification was based on comparison of mass spectra and retention times of authentic standards when available and with mass spectra in an essential oil library (Adams, 2009). However, in the majority of cases authentic standards were not available and it was therefore not possible to determine the stereochemistry of the identified compounds. Bergamotene, santalene, zingiberene and curcumene stereoisomers were assigned based on previous reports (Sallaud *et al.*, 2009; Bleeker *et al.*, 2011a). Compounds were quantified based on their abundance relative to the internal standard tetradecane and standard curves with β-caryophyllene and γ-terpinene. The average for the two to three replicates was calculated and used for hierarchical clustering applying the Bray–Curtis index, and the distance between samples determined with average linkage with the vegan package for R (Oksanen *et al.*, 2010).

### Trichome isolation and gene cloning

Stems and petioles from two to three fully grown plants from the same accession were pooled. Trichomes were collected by fast-freezing the tissue and scraping the frozen trichomes in liquid nitrogen. RNA was extracted using the RNeasy Plant Mini Kit (http://www.qiagen.com/) and cDNA prepared with the Transcriptor First Strand cDNA Synthesis Kit (http://www.roche-applied-science.com) using oligo(dT) as a primer. Amplification of full-length *TPS-e/f* cDNAs was performed using KOD DNA polymerase (http://www.emdchemicals.com/life-science-research) with conserved primers designed to previously published sequences (Sallaud *et al.*, 2009; Schilmiller *et al.*, 2009) (Table S3). Polymerase chain reaction (PCR) fragments were purified using the Wizard® SV Gel and PCR Clean-Up System (http://www.promega.com/) and cloned into the pCR®4Blunt-TOPO® vector (http://www.invitrogen.com/). Recombinant clones were verified by colony PCR and sequence analysis. Nucleotide sequences were deposited in Genbank under the following accession numbers JN990661–JN990694.

### DNA sequence analysis, multiple sequence alignments and phylogenetic analyses

DNA sequences were edited and assembled using Sequencher™ software version 4.8 (http://genecodes.com/). Sequence analysis was performed with MEGA version 5 (Tamura *et al.*, 2011) using MUSCLE to construct the alignments and the Minimum Evolution (ME) method for phylogenetic analysis. A bootstrap test was used to assess the reliability of the tree. Percent identity between nucleotide and predicted amino acid sequences of TPS enzymes was calculated using the alignment made by MUSCLE to calculate distance.

### Synthesis of codon optimized genes, recombinant protein expression and activity assays

Codon optimized versions of TPS-e/f enzymes that lacked the chloroplast targeting sequence but containing *Bam*HI and *Sal*I restriction enzyme recognition sequences at the 5′- and 3′-ends respectively, were synthesized by Genscript Corporation (http://www.genscript.com/). The synthetic gene was excised from pUC57 by digestion with *Bam*HI and *Sal*I and ligated into the pHIS8 vector previously linearized with the same enzymes. Recombinant clones were transformed into *E. coli* BL21 cells. A 5-ml log-phase culture of *E. coli* BL21 cells containing the expression vector was induced by addition of 0.1 mm IPTG. The induced cells were incubated with agitation at 25°C for 16 h, and then harvested by centrifugation. The cell pellet was resuspended in 1 ml extraction buffer (50 mm HEPES pH 8, 5% glycerol, 100 mm KCl, 7.5 mm MgCl_2_ containing 1 mm dithiothreitol) prior to sonication. Soluble proteins were harvested after centrifugation at 10 000 ***g*** for 20 min and the supernatant utilized for enzyme assays.

For the identification of reaction products, 5 μg of soluble protein were mixed with 10 μm of substrate, either GPP, NPP, *e,e*-FPP or *2z*,*6z*-FPP (http://www.echelon-inc.com/), in a 2-ml glass vial. Reactions were incubated for 30 min at 30°C, and the headspace was collected for 5 min with a 65-μm polydimethylsiloxane-divinylbenzene solid-phase microextraction fiber (http://www.sigmaaldrich.com/analytical-chromatography.html). For comparison of enzyme products with trichome terpene profiles, stem trichomes were collected from greenhouse-grown plants and an aliquot extracted with hexane containing 1 ng μl^−1^ of tetradecane as internal standard. Enzyme assays containing 40 μm of substrate and 50 μg of soluble protein in 100 μl were overlaid with two volumes of hexane. After 1 h at 30°C, the vials were vigorously mixed and centrifuged to separate the organic layer. The reactions were frozen at −80°C, and the hexane layer transferred to a new vial. GC–MS analysis was performed as described previously (Schilmiller *et al.*, 2010a).

### Analysis of geographical distribution and climate data

When available, latitude and longitude information for the original collection site of *S. habrochaites* accessions was obtained from the TGRC (Table S1). Locations were mapped using DIVA-GIS 7.5.0.0 software (http://www.diva-gis.org/). Climate and elevation data were obtained from WordClim v1.3 at 5 min resolution (Hijmans *et al.*, 2005). Correlation with volatile terpene profiles was performed using R.

## SUPPORTING INFORMATION

**Figure S1.** Terpene abundance and composition in a subset of *S. habrochaites* accessions.

**Figure S2.** Alignment of representative TPS-e/f proteins.

**Figure S3.** Phylogeny of TPS20-related proteins identified in 23 *S. habrochaites* accessions.

**Figure S4.** Comparison of recombinant TPS-e/f activities and trichome terpene profiles.

**Figure S5.** Characterization of major terpenes synthesized by ShPHS1 and ShLMS.

**Figure S6.** Characterization of minor peaks identified in TPS enzyme activity assays.

**Figure S7.** Heterogeneity in accession LA2104.

**Table S1.** *S. habrochaites* accessions, their geographic distribution and associated climate data.

**Table S2.** Mono- and sesquiterpenes terpenes identified in *S. habrochaites* trichomes.

**Table S3.** Primers used in this study.

Please note: As a service to our authors and readers, this journal provides supporting information supplied by the authors. Such materials are peer-reviewed and may be re-organized for online delivery, but are not copy-edited or typeset. Technical support issues arising from supporting information (other than missing files) should be addressed to the authors.
