# Supplementary material for: Evolution of TPS20-related terpene synthases influences chemical diversity in the glandular trichomes of the wild tomato relative Solanum habrochaites
Source: Plant J. 2012 Jun 22;71(6):921–35. doi: 10.1111/j.1365-313X.2012.05040.x (PMC3466413; doi:10.1111/j.1365-313X.2012.05040.x)
Supplement: Supplementary file 9 [file tpj0071-0921-SD9.doc]

**Supplementary table 1.** *S. habrochaites* accessions, their geographic distribution and associated climate data.

**Supplementary table 2.** Mono- and sesquiterpenes terpenes identified in *S. habrochaites* trichomes.

**Supplementary table 3.** Primers used in this study.

**Supplementary figure 1. Terpene abundance and composition in leaf trichomes of a subset of *S. habrochaites* accessions.**

The chemical composition of a subset of 27 *S. habrochaites* accessions that represent the diversity observed in the 79 accession screen was verified by re-growing the plants and calculating the terpene content using standard curves made with γ-terpinene and β-caryophyllene for the quantification of mono- and sesquiterpenes, respectively. (a), terpenes broken down individually; (b) total amount of monoterpenes (gray bars) and sesquiterpenes (black bars) is shown as well as the standard deviation.

**Supplementary figure 2. Protein alignment of representative TPS-e/f from each clade.**

The DDXXE and NSE/DTE motif are marked. The amino acid deletion present in the clade C sequences is indicated (▼ ).Similar residues are highlighted in gray, divergent residues in black.

**Supplementary figure 3. Phylogeny of TPS20-related proteins identified in 23 *S.***

***habrochaites* accessions.** An unrooted tree constructed with the ME method using 37 sequences from various *S. habrochaites* accessions together with SlPHS1 (FJ797957), SpPHS1 (JN412071), and ShSBS (ACJ38409). Two major groups designated M (monoterpene) and S (sesquiterpene), which subdivide into Clade A, B and C; and Clade D and E, respectively. Bootstrap values above 50 are shown. Colored symbols indicate the chemical group (defined in

Figure 2) from which each sequence was isolated.

**Supplementary figure 4. Catalytic activities of recombinant TPS compared to terpene profile of stem trichomes.**

ShPHS1 obtained from LA2100 (a), ShLMS from LA2812 (b), ShPIS from LA2107 (c), ShSBS from LA1393 (d) and ShZIS from LA2167 (e) expressed in *E. coli* and assayed with NPP (a through c) or *2z,6z*-FPP (d and e) as substrates. Extracted ion chromatograms for *m/z* 93 are shown. 1, α-pinene; 2, δ-2-carene; 3, α-phellandrene; 4, limonene and β-phellandrene; 5, limonene; 6, β-pinene; 7, endo-α-bergamotene; 8, (+)-α-santalene; 9, γ-elemene; 10, (+)-endo-β-bergamotene; 11, (-)-exo-α-bergamotene; 12, (-)epi-β-santalene;13,unknown; 14, *R*-curcumene; 15, 7-epizingiberene; 16, β-sesquiphellandrene

**Supplementary figure 5. Characterization of major terpenes synthesized by ShPHS1 and**

**ShLMS.**

(a) Chromatogram of the products obtained by incubation of ShPHS1 with NPP. The predominant peak (*) is a mixture of β-phellandrene and limonene that elute at the same time. (b) Mass spectra of limonene and β-phellandrene identified in *in vitro* assays with ShPHS1. (c) Chromatogram of the products obtained by incubation of ShLMS with NPP. The predominant

peak (*) is limonene. (d) Mass spectra of limonene identified in *in vitro* assays with ShLMS.

**Supplementary figure 6.** Characterization of minor peaks identified in TPS enzyme activity assays. Headspace collection of volatiles produced by recombinant TPS from LA1393 (ShSBS)

(a), LA2167 (ShZIS) (b), LA2100 (ShPHS1) (c), LA2812A (ShLMS) (d), LA2107 (ShPIS) (e) expressed in *E. coli* and assayed with *2z,6z*-FPP as a substrate. Reactions with the empty vector are included as a control (f). Extracted ion chromatograms for *m/z* 93 are shown. 9, endo-α- bergamotene; 10, (+)-α-santalene; 11, (-)-exo-α-bergamotene; 12, (-)epi-β-santalene; 13, (+)- endo-β-bergamotene; 14, (*Z*)-β-farnesene; 15, β-bisabolene; 16, β-sesquiphellandrene.; 17, unknown; 18, *R*-curcumene; 19, 7-epizingiberene; 20, unknown; 21, unknown. Numbering of compounds is the same as that reported in Figure 6.

**Supplementary figure 7. Heterogeneity in accession LA2104.**

Three individual 3-week old plants were used for collection of volatiles. Extracted ion chromatogram at *m/z* 93 shows that the second plant has a more complex terpene profile, with products from ShPHS1and ShZIS detected.
